# Registered reports: an early example and analysis

**DOI:** 10.7717/peerj.6232

**Published:** 2019-01-16

**Authors:** Richard Wiseman, Caroline Watt, Diana Kornbrot

**Affiliations:** 1 Department of Psychology, University of Hertfordshire, Hatfield, United Kingdom; 2 School of Philosophy, Psychology and Language Sciences, University of Edinburgh, Edinburgh, United Kingdom

**Keywords:** Psychology, Replication, Registered reports, Methodology, Publication bias

## Abstract

The recent ‘replication crisis’ in psychology has focused attention on ways of increasing methodological rigor within the behavioral sciences. Part of this work has involved promoting ‘Registered Reports’, wherein journals peer review papers prior to data collection and publication. Although this approach is usually seen as a relatively recent development, we note that a prototype of this publishing model was initiated in the mid-1970s by parapsychologist Martin Johnson in the *European Journal of Parapsychology (EJP)*. A retrospective and observational comparison of Registered and non-Registered Reports published in the *EJP* during a seventeen-year period provides circumstantial evidence to suggest that the approach helped to reduce questionable research practices. This paper aims both to bring Johnson’s pioneering work to a wider audience, and to investigate the positive role that Registered Reports may play in helping to promote higher methodological and statistical standards.

## Background

In 2011, Daryl Bem published a paper in the *Journal of Personality and Social Psychology* describing the results from nine experiments that appeared to support the existence of psychic ability (Bem, 2011). The high-profile nature of the journal, combined with the controversial findings, resulted in Bem’s paper attracting a considerable amount of attention within both academia and the media (Carey, 2011; Halliwell, 2011). Several academics were critical of Bem’s paper, with researchers subsequently reporting a failure to replicate his experiments (Ritchie, Wiseman & French, 2012), commenting on the a priori unlikelihood of psychic ability existing (Wagenmakers et al., 2011) and questioning the validity of the original studies (Wagenmakers et al., 2011; Alcock, 2011; Francis, 2012; Schimmack, 2012). This latter strand of criticism focused on a variety of methodological and statistical issues, including the lack of a detailed analysis plan, selective reporting of data, post hoc analyses being presented as confirmatory findings, and the incomplete description of experimental procedures.

Some commentators noted that many of the criticisms aimed at Bem’s work could also apply to research within mainstream behavioral science (LeBel & Peters, 2011). Additional work on this topic identified several ’questionable research practices’ (QRPs), including the failure to publish null studies (creating the so-called ‘file drawer problem’), the alteration of hypotheses after data collection (often referred to as ‘Hypothesizing After the Results are Known’ or ‘HARKing’), and the fishing around in data for significant findings (‘p-hacking’: see, e.g., John, Loewenstein & Prelec, 2012; Neuroskeptic, 2012). This work, combined with the results from a large-scale initiative questioning the replication rates of some well-regarded psychological effects (Open Science Collaboration, 2015), laid the foundations for the recent ‘replication crisis’ in psychology (Pashler & Wagenmakers, 2012).

Some researchers have begun to address issues surrounding poor replication rates by developing procedures to help minimise QRPs (Nosek, Spies & Motyl, 2012). One of the most popular approaches involves encouraging experimenters to describe their hypotheses and planned analyses prior to data collection (Wagenmakers et al., 2012; Van ‘t Veer & Giner-Sorolla, 2016). This concept, known as ‘pre-registration’, helps minimize several of the most important QRPs, including the selective reporting of studies, HARKing and p-hacking.

There are two main forms of study registration. The first approach involves experimenters producing a description of their intended study (including the number of participants, hypotheses and planned analyses) and then submitting this information to some form of trusted study registry such as the Open Science Framework (Nosek et al., 2018).

Study registries have operated for a long time. The first registries were established by medical researchers in the 1960s, and were originally designed to help experimenters recruit participants for clinical trials rather than prevent QRPs (Dickerson & Rennie, 2003). From the mid-1980s onwards, however, medical researchers began to recognize the importance of the issues surrounding the non-publication of null results (Simes, 1986; Easterbrook et al., 1991) and so developed study registries explicitly designed to tackle the problem.

Kaplan & Irvin (2015) recently demonstrated the need for such registries. In 2000, the National Library of Medicine at the National Institutes of Health required medical researchers to preregister key aspects of their studies (including experimental protocol, sample sizes, plans for handling missing data, and statistical analyses). Kaplan & Irvin compared study outcomes before and after the mandatory need for preregistration, examining studies that had investigated the impact of drugs and dietary supplements on cardiovascular disease. Remarkably, 57% of the studies published prior to 2000 reported a significant effect, compared to just 8% of studies published after the introduction of mandatory preregistration.

In 2008, the Neuroskeptic blog (Oct. 25 2008; Nov. 3, 2008) discussed the need for preregistration within psychology. To our knowledge, psychology’s first formal study registry (reviewing all submissions and making them irreversibly public) was launched in 2012 at the University of Edinburgh and focused on parapsychological research (Watt, 2012; Watt & Kennedy, 2015). In 2013, Jona Sassenhagen from the University of Marburg was the first researcher to preregister a mainstream psychological study, albeit using a registry designed to log clinical studies (Neuroskeptic, Feb 3 2013). Since then, several online platforms have been created for study registration within the behavioral sciences, including the Open Science Framework (https://osf.io), ‘As Predicted’ (https://aspredicted.org) and The American Economic Association’s Registry for Randomized Controlled Trials (https://www.socialscienceregistry.org).

The second type of preregistration is journal-based, and involves investigators producing a complete description of their intended study (including experimental rationale, hypotheses, method and planned analyses) and then submitting this report for peer review prior to data collection. If the submission is accepted, the authors are guaranteed publication regardless of study outcome. Chambers (2017) coined the phrase ‘Registered Reports’ (RRs) to describe this procedure.

There are several ways in which RRs can help to improve the quality of research. As with all forms of pre-registration, RRs require experimenters to pre-specify several aspects of their study (including planned hypotheses, number of participants and intended analyses) and so help to prevent publication bias, p-hacking and HARKing. In addition, RRs require investigators to describe their study rationale and methodological procedures, thus presenting referees with an opportunity to help improve the theoretical basis and design of an experiment prior to data collection.

When tracing the historical roots of this idea, Chambers (2017) has noted that Rosenthal (1966), Walster & Cleary (1970) and Newcombe (1987) all outlined early versions of this idea, primarily in an attempt to combat publication bias. Similarly, Kupfersmid (1988) suggested that peer review should be conducted prior to data analysis, noting that this would help prevent publication bias and p-hacking. Weiss (1989) also recommended that a paper be reviewed prior to data collection, but suggested that this would help to prevent researchers wasting their time running poor quality studies, rather than minimising QRPs. Unfortunately, psychology journals at the time failed to adopt the procedure.

In 2012, Chambers was invited to join the Editorial Board of *Cortex*, and suggested that the journal help prevent QRPs by encouraging researchers to submit papers for review prior to data collection (Chambers, 2017). In 2013, *Cortex* adopted Chambers’ suggestion (Chambers, 2013), and Chambers & Munafo (2013) published an open letter calling for other journals to adopt the same approach. This letter was signed by a large number of psychologists and helped attract attention to the notion of RRs. Currently, over a hundred journals now accept this form of submission (Center for Open Science, 2018).

Understandably, attempts to outline the historical roots of RRs have tended to focus on previous research within mainstream psychology and the adoption of the procedure by *Cortex* in 2013. However, many academics are unaware that a prototype version of RRs was implemented in the mid 1970s by a little-known parapsychology journal, and that this pioneering publication policy ran successfully for many years.

In 1973, psychologist Martin Johnson was officially appointed as a professor of parapsychology at the University of Utrecht (Schouten, 1988 –1989; Parker & Mörck, 2011). Johnson remained in post until 1986 and passed away in 2011.

Due to its controversial subject matter, parapsychology has traditionally attracted a considerable amount of critical attention. During the mid-1970s much of this attention focused on identifying potential methodological and statistical shortcomings, and developing ways to help minimize these issues. Much of this debate anticipated the present-day work into QRPs in mainstream psychology, and involved detailed discussions on the impact of post hoc analyses (Wiklund, 1977) and publication bias (Rhine, 1975; Johnson, 1976).

In November 1974, Johnson gave his inaugural professorial address at the University of Utrecht and subsequently published some of the key points from the talk in a journal article (Johnson, 1975). In this article, Johnson argued that it was vital to minimise possible methodological issues in both parapsychology and mainstream psychology. He then outlined three ways of conducting research and explored the degree to which each was open to bias. The first approach simply involved a researcher carrying out an experiment on their own whilst the second approach involved them working as part of a team. As such, both approaches were relatively informal in nature and so open to several QRPs. However, the third approach described by Johnson was far more rigorous and was explicitly designed to prevent several methodological and statistical problems:

“... according to the philosophy of this model, the experimenter should define his problem, formulate his hypotheses, and outline his experiment, prior to commencing his study. He should write his manuscript, stating at least essential facts, before carrying out his investigation. This manuscript, in principle only lacking data in the tables, presentation of results, and interpretation of results, should be sent to one or more editors, and the experimenter should not initiate his study until at least one of the editors has promised to publish the study, regardless of the outcome of the experiment. In this way we could avoid selective reporting. Furthermore the experimenter will not be given the opportunity to change his hypotheses in such a way that they “fit” the outcome of the experiment.” (Page 41)

In short, in 1974, Johnson outlined many of the key attributes now associated with RRs, including investigators describing important aspects of a study prior to data collection, the reviewing of this report, and the guarantee of publication regardless of study outcome.

Johnson then teamed up with another parapsychologist and member of the Utrecht Psychology Department, Sybo Schouten, and together they launched The *European Journal of Parapsychology* (*EJP*). This little-known journal was primarily designed to publish experimental work testing the possible existence of psychic ability. In Volume 1:1 (November 1975), the Editors outlined their preference for researchers to submit papers prior to data collection (Johnson & Schouten, 1975). This initial volume also contained the paper based on Johnson’s inaugural address. Volume 1:2 (May 1976) contained another article by Johnson about the importance of this policy for combatting the non-publication of null results (Johnson, 1976). Volume 1:3 (November 1976) contained the first formal statement describing the journal’s publication policy and noted:

“A hallmark of the *European Journal of Parapsychology* is the attempt to avoid selective reporting, that is, the tendency to bury ’negative’ results and only to publish studies that ’turn out’. To avoid turning the journal into a graveyard for all ’unsuccessful’ studies, we require that the acceptance or rejection of a manuscript should take place prior to the phase when the experimental data are collected. The quality of the design and methodology and the rationale of the study should be judged as per se more important than the level of significance of the outcome of the study. As a practical rule, we advise a contributor of an article to submit a design of his planned study before the study is actually carried out. The rationale of the study should be stated, as well as all the hypotheses related to it. Furthermore one should try to specify the number of subjects, the number of trials, etc., plus the type of statistical methods one plans to use for one’s evaluation. Priority will be given to the publication of studies which fulfil the above-stated publication policy.”

As such, the *EJP* editors embraced the underlying ethos of RRs (including the importance of publishing both positive and negative results, and judging the quality of research prior to data collection) and made initial attempts to devise a system that encapsulated many of the key attributes of RRs (including encouraging researchers to submit a document specifying their experimental protocol, sample size, number of trials, rationale and hypotheses, and data analyses). It should be noted, however, that the *EJP* editorial guidelines did not involve many of the more elaborate checks and balances associated with many modern-day systems for RRs (including, for instance, researchers having to complete templates that require them to pre-specify key information; editors, referees and authors working together to form an ‘in-principle acceptance’ of the study; a second round of peer reviewing post data collection; logging uncompleted or withdrawn studies; the storage and publication of researchers’ initial documentation). In addition, as the initial documentation submitted to the *EJP* by researchers was not, to our knowledge, retained or published, it isn’t possible to retrospectively judge the degree to which researchers adhered to the editorial guidelines. These shortcomings aside, it’s clear that the EJP editors developed and carried out a prototypical version of modern-day systems for RRs.

This publication statement then appeared in every issue of the *EJP* from 1976 to 1992. In 1992, following the closure of Utrecht’s parapsychology laboratory, the *EJP* editorship transferred to the Koestler Parapsychology Unit (University of Edinburgh), and a slightly modified version of the publication policy (albeit one still emphasising an openness to reviewing manuscripts prior to data collection) appeared between 1992 and 2000. The final RR was published in the EJP in Volume 9 (1992-1993). In 2000, the *EJP* editorship transferred to the University of Stockholm and the publication policy no longer referred to RRs.

For about 17 years (1976 to 1993), the *EJP* published a mixture of RRs and non-RRs. In addition to playing an important, and little known, role in the history of RRs, this unique database presents an opportunity to conduct an exploratory and retrospective study assessing the impact of RRs on study outcome. Given that RRs were designed to reduce QRPs, it was hypothesised that RRs would contain a lower proportion of statistically significant results than non-RRs. Additional analyses aimed to explore whether such a finding could be due to two alternative explanations (namely, whether any differences were due to the RRs and non-RRs involving different types of studies or being conducted at different times). All analyses were exploratory, and all data exclusions and measures have been reported.

## Method

### Design: this study employed a retrospective observational design

#### Dataset and coding

The dataset consisted of all experimental papers that tested for the existence of psychic ability in the issues of the *EJP* between the publication of the first and last RR (Volume 1:3 [1976] to Volume 9 [1992–1993]). This dataset contained 63 papers reporting 110 experiments. A research assistant made two copies of each paper and then removed the front page of the paper (which contained a footnote indicating whether the paper was a RR), randomised the order of the papers, and presented them to two of the authors (RW and CW) for blind coding.

Experiments were rejected if they didn’t contain at least one formally stated hypothesis (*N* = 4), or if the authors described a methodological artefact that they believed undermined the entire experiment (*N* = 15). Examples of the latter category included: (i) experimenters attempting to investigate alleged psychic influence upon seed growth, but noting that they had not ruled out possible ‘normal’ influences due to non-blind planting, handling, and measurement (Solfvin, 1982); and (ii) researchers conducting a series of pilot studies in which groups of participants were asked to psychically determine the nature of a hidden target, and noting that their data was non-independent and so couldn’t be assessed in a meaningful way (Blackmore, 1981). Three papers were removed from the database because all of their experiments had been rejected.

The remaining experiments were assigned a unique experiment identity number (ExperimentID) and coded on the following variables:

***N:*** The number of formal hypotheses tested. Hypotheses that were clearly labelled as exploratory, post hoc or informal were excluded.

***H:*** The number of hypotheses supported. The experiments involved in the analysis were conducted from the mid 1970s to the mid 1990s and at the time researchers tended to focus on whether their findings were statistically significant rather than on effect sizes. As a result, several papers contained a paucity of statistical information and a few even simply stated whether the results were, or were not, significant. Therefore, it was decided to employ the metric that was reported in every paper, and one that would have been most relevant to researchers at the time the studies were conducted, namely, whether the analysis testing the hypothesis was reported to be statistically significant.

#### Topic

Parapsychological experiments are traditionally seen as testing one of two types of alleged psychic ability: Extra-Sensory Perception (ESP: The alleged awareness of information about external events not gained through the traditional senses or deducible from previous experience) and Psychokinesis (PK: The alleged mental influence of a physical or biological system without physical interaction). Some researchers have argued that the effects allegedly obtained in ESP experiments are more robust than those in PK studies (see, e.g., Jahn et al., 2000; Bösch, Steinkamp & Boller, 2006). To help assess whether any differences in the proportion of significant findings in RRs and non-RRs might be due to the two sets of studies focusing on different topics, each study was coded as testing either ‘ESP’ or ‘PK’.

#### Time

It was possible that the *EJP* papers were less likely to report significant effects over time (perhaps due to the ongoing identification and elimination of methodological artifacts) and that the RRs tended to be published in later journals. To help examine this possibility, the Journal Issues were numbered chronologically from ‘1′(Volume 1:3) to ‘23′(Volume 9).

Each coder independently rated each paper, and then any areas of disagreement were resolved prior to breaking the blind. After the coding had been completed, the Registration Status of each experiment was coded as ‘RR’ or ‘non-RR’.

## Results

The final dataset contained 60 papers: 25 RRs and 35 non-RRs. The RRs described 31 experiments that tested 131 hypotheses, and the non-RRs described 60 experiments that tested 232 hypotheses.

28.4% of the statistical tests reported in non-RRs were significant (66/232: 95% CI [21.5%–36.4%]); compared to 8.4% of those in the RRs (11/131: 95% CI [4.0%–16.8%]). A simple 2 × 2 contingency analysis showed that this difference is highly statistically significant (Fisher’s exact test: *p* < .0005, Pearson chi-square=20.1, Cohen’s *d* = .48). A Generalized Linear Model analysis (Probit Model: Response Variable; H/N Predictor; Registration Status: Random Factor; ExperimentID) yielded a significant effect of Registration Status (*F*(1, 89) = 16.3, *p* = .0001, Cohen’s *d* = .43). To examine whether this effect could be due to RRs and non-RRs differentially examining the alleged existence of ESP or PK, ‘Topic’ was added to the Generalized Linear Model and had no significant improvement (*F*[1, 87] = .56, *p* = .45; for Topic by Registration Status: *F*[1, 87] = 1.79, *p* = .18). In addition, the variable ‘Time’ was added as a continuous marker but yielded no significant effects (*F*[1, 87] = 1.01, *p* = .32; for journal issue by Registration Status: *F*[1, 87] = .14, *p* = .71). These findings suggest that the difference in the proportion of significant findings reported in RRs and non-RRs was not due to the two sets of studies investigating different topics, or improved methodology over time corresponding with fewer significant outcomes.

## Discussion

Researchers have recently started to use RRs as a way to minimise QRPs within the behavioral sciences. Much of the literature describing the historical roots of RRs has focused on previous work within mainstream psychology and the key role played by *Cortex* in 2012. However, in the mid-1970s parapsychologist Martin Johnson proposed a prototypical version of RRs. Johnson and parapsychologist Sybo Schouten then launched the *EJP* and explicitly encouraged researchers to submit RRs. Over the next 17 years, this journal published a mix of RRs and non-RRs.

Most present-day systems for RRs usually involve several stages, including an editorial pre-review, refereeing of the paper prior to data collection, and an additional refereeing of the completed paper. In contrast, the procedure created by Johnson only involved a single round of peer review prior to data collection. In addition, whereas modern-day researchers submitting a RR are asked to explicitly pre-specify a series of details about their study, the *EJP* publication policy was more relaxed and simply urged authors to present their rationale, hypotheses, number of subjects and trials, planned statistical analyses etc. (unfortunately, to our knowledge, the initial documentation submitted to the *EJP* wasn’t archived and so it isn’t possible to assess the detail or accuracy of the submitted material). Nevertheless, clearly Johnson’s approach was similar to present-day systems for RRs, and pre-dated those systems by about forty years.

The *EJP*’s mix of RRs and non-RRs allowed us to assess the relationship between RRs and study outcome. Compared to non-RRs, RRs were significantly less likely to contain statistically significant results. In addition, there was no evidence to suggest that this effect was due to differences in the parapsychological topics under investigation, or to the two sets of studies being carried out during different time periods. As such, these results are consistent with the notion that RRs helped reduce QRPs (which, in turn, reduced the presence of Type 1 errors), and are in line with similar work reported in the medical literature (Kaplan & Irvin, 2015). However, the *EJP* studies were not randomly allocated to condition, so the RRs and non-RRs may have varied on several other factors (including, for example, study design, power, and methodological quality), therefore it is possible that these factors may be responsible for the observed effect. As a result, the findings should be seen as circumstantial, rather than definitive, evidence for the notion that RRs help prevent QRPs.

Parapsychologists investigate the possible existence of phenomena that, for many, have a low a priori likelihood of being genuine (see, e.g., Wagenmakers et al., 2011). This has often resulted in their work being subjected to a considerable amount of critical attention (from both within and outwith the field) that has led to them pioneering several methodological advances prior to their use within mainstream psychology, including the development of randomisation in experimental design (Hacking, 1988), the use of blinds (Kaptchuk, 1998), explorations into randomisation and statistical inference (Fisher, 1924), advances in replication issues (Rosenthal, 1986), the need for pre-specification in meta-analysis (Akers, 1985; Milton, 1999; Kennedy, 2004), and the creation of a formal study registry (Watt, 2012; Watt & Kennedy, 2015). Johnson’s work on RRs provides another striking illustration of this principle at work.

Finally, the analysis of *EJP* papers based on RRs revealed that around 8.4% of the findings were statistically significant, compared to the 5% expected by chance alone. Although significant findings reported in RRs represent higher quality evidence than those reported in non-RRs, this result is not compelling evidence for the existence of psychic ability as the experiments may have contained other non-obvious methodological shortcomings, such as issues regarding sensory leakage and poor randomisation (Milton & Wiseman, 1997).

## Conclusion

Over the past few years, many psychologists have focused attention on the reduction of questionable research practices and the promotion of replication rates. Much of this work has involved encouraging investigators to preregister their experiments as a RR. The idea of RRs within the behavioural sciences is seen as a relatively recent development, and is often perceived as having first been adopted by journals around 2012.

In fact, a prototype of this publishing model was initiated in the mid-1970s by parapsychologist Martin Johnson, and ran many years in the *European Journal of Parapsychology*. An empirical comparison of RRs and non-RRs provides circumstantial evidence to suggest that the approach may have helped to reduce questionable research practices.

It seems fitting that the final word goes to one of the researchers involved in *EJP’s* pioneering publication policy. Thirty years ago, Schouten (1988–1989) noted that the innovative policy that he had helped to create might act as a useful testing ground for a procedure that might, one day, prove valuable to mainstream science:

“Especially in a disputable area such as parapsychology it is important to face all kinds of challenges with an open mind and to introduce new and better ways of doing things. We might well compensate for our, in many eyes, suspicious subject matter by setting new and better standards in areas of the scientific process, as for instance methodology or editorial policies.” (Page 101)

It is pleasing to see that Schouten’s thoughts have now become a reality.

##  Supplemental Information

10.7717/peerj.6232/supp-1 Supplemental Information 1 Data, cookbook and analysis scripts

## Additional Information and Declarations

### Data Availability

The following information was supplied regarding data availability: The list of coded journal articles is provided, along with cookbook and analyses scripts as a Supplemental File.
